# PACE AND FFS MEDICARE OUTCOMES BEFORE AND DURING COVID-19: A PROPENSITY SCORE MATCHED ANALYSIS

**DOI:** 10.1093/geroni/igad104.3513

**Published:** 2023-12-21

**Authors:** Morgan Perry, Samuel Obbin, Stephen McCall, Joseph Dorris, Michael Nardone, Ani Turner, Christine Stanik

**Affiliations:** University of Michigan, Ann Arbor, Michigan, United States; Altarum, Ann Arbor, Michigan, United States; Altarum, Ann Arbor, Michigan, United States; Altarum, Ann Arbor, Michigan, United States; The Nardone Group LLC, Philadelphia, Pennsylvania, United States; Altarum, Ann Arbor, Michigan, United States; Arbor Research Collaborative, Ann Arbor, Michigan, United States

## Abstract

We compared outcomes of Program of All-Inclusive Care for the Elderly (PACE) participants and fee-for-service (FFS) Medicare, dually eligible individuals using a retrospective cohort study with propensity score matching on demographic, socioeconomic, and participant-specific acuity level variables. PACE participants in 132 PACE centers nationwide who were dually eligible, enrolled during the study period, and did not have end-stage renal disease were included. Over 80% of eligible PACE participants were matched with FFS beneficiaries during each period, bringing many covariates closer between groups. We assessed differences during each period using Wilcoxon signed rank test and over time using differences-in-differences analyses. PACE participants spent significantly more time in the community and less time in nursing homes and hospitals (all p< 0.01). PACE participants had fewer COVID-19 diagnoses and hospitalizations per member per month (all p< 0.01). Due to the blinding of data, we could not determine definitively which performed better in limiting severe pressure ulcers. However, the results indicate PACE performed better when the mean assumption for blinded data is used. For all-cause mortality, PACE participants continued to perform better than FFS both before and during the pandemic (all p< 0.01), but the rates were closer during the pandemic periods. The flexibility in the PACE model and its person-centered care delivery approach may have contributed to the positive outcomes compared to FFS before and during the pandemic. We should consider how best to leverage the PACE model of care to support older adults to remain in their homes and avoid institutional settings.

